# Rising trends in infant ER encounters for food-induced allergic reactions in the era of early allergenic food introduction

**DOI:** 10.1016/j.jacig.2025.100637

**Published:** 2025-12-22

**Authors:** Aaron Chin, Ali Doroudchi, Derek C. Pham, Nicholas J. Jackson, Maria I. Garcia-Lloret

**Affiliations:** a Division of Pediatric Allergy/Immunology and Rheumatology, Department of Pediatrics, University of California Los Angeles School of Medicine, Los Angeles, Calif; b Division of General Internal Medicine and Health Services Research, Department of Medicine at the University of California Los Angeles, Los Angeles, Calif

**Keywords:** Allergenic food, anaphylaxis, early allergen introduction, infant reaction, emergency department, epinephrine, guideline adoption

## Abstract

**Background:**

Allergic reactions to food are a leading cause of pediatric emergency room (ER) visits. National guidelines now recommend early introduction of allergenic foods; however, whether these changes have affected ER encounters remains poorly understood.

**Objective:**

Our aim was to observe trends in ER encounters for food-induced reactions (FIRs), including food-induced anaphylaxis (FIA), among children aged 0 to 5 years between 2013 and 2024.

**Methods:**

We performed a retrospective analysis of ER visits at the University of California Los Angeles from 2013 to 2024. FIRs were identified by using International Classification of Diseases codes and stratified by age (0-1 vs 2-5 years). Logistic regression assessed annual trends. Multivariate logistic regression was used to compare postguideline years (2022-2024) with preguideline years (2013-2016), adjusting for clinical and demographic variables.

**Results:**

Of 67,059 ER visits, 350 FIRs and 182 FIA visits were identified. FIR and FIA annual rates increased significantly over time in infants aged 0 to 1 years (odds ratio [OR] = 1.15 [*P* = .005] and OR = 1.27 [*P* = .002]). In children aged 2 to 5 years old, neither FIR nor FIA rates increased. Refractory anaphylactic reactions did not change in either group. In multivariate analysis, infants in the postguideline period had more than twice the odds of a FIR during the preguideline years (OR = 2.21 [95% CI = 1.37-3.55]), whereas no change was observed in the 2- to 5-year age group (OR = 0.82 [95% CI = 0.55-1.2]).

**Conclusion:**

ER visits for food reactions have continued to rise at a steady rate over time among infants. These findings underscore the need for additional research on adoption, parental guidance, and long-term impact of early allergen introduction.

## Introduction

Food allergy is a leading cause of anaphylaxis in children visiting emergency rooms (ER).^1^, ^2^, ^3^ An estimated 8% of children have food allergy, and 40% have experienced food-related severe reactions, including anaphylaxis.^4^ In 2015, the LEAP (Learning Early About Peanut Allergy) randomized controlled trial demonstrated that early peanut introduction in high-risk infants could prevent peanut allergy.^5^ Alongside LEAP, trials such as EAT (Enquiring about Tolerance) and supporting meta-analyses have demonstrated that early introduction of peanut and egg reduces the risk of developing allergy to these foods, with evidence of sustained benefit through at least age 5 years.^6^, ^7^, ^8^ These findings led to a global shift in infant feeding guidelines, with major bodies, including the National Institute of Allergy and Infectious Diseases (2017), Asthma Action Plan (2019), and American Academy of Allergy, Asthma & Immunology (2021), recommending food allergen introduction at age 4 to 6 months.^9^^,^^10^ Using available electronic health record data, we sought to examine correlations in trends in ER visits for food-related reactions in children aged 0 to 5 years following guideline-based changes in the recommendation of early allergen introduction.

## Results and discussion

**Fig 1:**
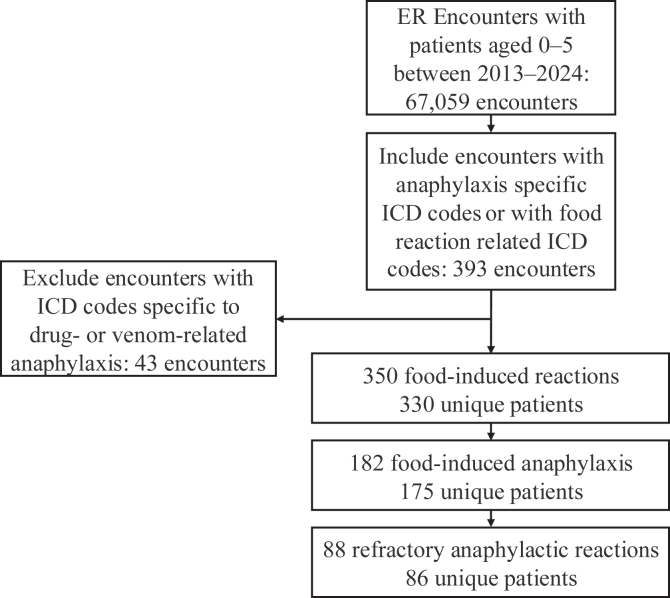
Flow diagram of ER encounters in children aged 0 to 5 years at the University of California Los Angeles from 2013 to 2024. Encounters were identified by using ICD codes for anaphylaxis (T78.0∗, T78.2∗, 995.6, and 995.0) or food-induced allergic reactions (T78.1∗, Z91.01∗, and V15.01-V15.05). Encounters with ICD codes specific to drug- or venom-related anaphylaxis (T63∗, Z91.03∗, T80.5∗, T88.6∗, 989.5, V15.06-V15.09, and 995.2) were excluded. Anaphylaxis encounters were defined as food-related visits with ICD codes and documented epinephrine use. Refractory anaphylactic reactions were defined as those requiring multiple epinephrine doses or hospitalization.

Of the 67,059 ER encounters at the University of California Los Angeles from 2013 to 2024 for patients aged 0 to 5 years, 393 encounters were identified as having anaphylaxis- or food-specific International Classification of Diseases (ICD) codes. Of those encounters, 43 were excluded owing to the presence of drug- or venom-related reactions. A total of 350 food-induced reaction (FIR) encounters involving 330 unique patients were included (Fig 1). Infants (aged 0-1 years) accounted for 46% of encounters, and children aged 2 to 5 years accounted for 54% (Table I). Of these encounters, 185 (52%) met the criteria for food-induced anaphylaxis (FIA), defined as either use of an anaphylaxis-based ICD code or use of epinephrine during the encounter. Across the study period, the proportion of FIR encounters that were FIA reactions was higher in children aged 2 to 5 years than that in infants (63% vs 40% [*P* < .001]), whereas the proportion with refractory reactions did not differ significantly between the 2 groups (28% vs 23% [*P* = .35]). Most patients (73%) were discharged, whereas 25% were hospitalized (*P* = .56). Epinephrine was used in 24% of encounters—more frequently in children aged 2 to 5 years than in infants (28% vs 19% [*P* = .046]), with repeat dosing in 7% of cases. Eczema was present in 46% of patients overall, with no reliable difference in the rates across age groups (41% vs 50% [*P* = .13]). Preexisting epinephrine prescriptions were more common in the 2- to 5-year age group (27% vs 20% [*P* = .001]), and prior allergy evaluations were also more frequent (40% vs 15% [*P* < .001]).

**Table I:** Demographics and encounter details

Demographics/details	All (N = 350)	Age 0-1 y (n = 162)	Age 2-5 y (n = 188)	*P* value
Unique patients, no.	330	156	179	
Sex, no. (%)				.011
Female	136 (38.9)	75 (46.3)	61 (32.4)	
Male	214 (61.1)	87 (53.7)	127 (67.6)	
Race/ethnicity, no. (%)				.13
White	125 (35.7)	62 (38.3)	63 (33.5)	
Asian	70 (20.0)	27 (16.7)	43 (22.9)	
Hispanic or Latino	46 (13.1)	27 (16.7)	19 (10.1)	
Black or African American	12 (3.4)	4 (3.5)	8 (4.3)	
Middle Eastern or North African	3 (0.9)	1 (0.6)	2 (1.1)	
Other or multiethnic	49 (14.0)	26 (16.0)	23 (12.2)	
Unspecified	45 (12.9)	15 (9.3)	30 (16.0)	
ER disposition, no. (%)				.56
Discharged	257 (73.4)	115 (71.0)	142 (75.5)	
Admitted/transferred	88 (25.1)	45 (27.8)	43 (22.9)	
Left without being seen	5 (1.4%)	2 (1.2)	3 (1.6)	
Epinephrine use, no. (%)				
Preexisting epinephrine prescription∗	71 (20.3)	20 (20.3)	51 (27.1)	.001
Epinephrine used in encounter	83 (23.7)	30 (18.5)	53 (28.2)	.046
Multiple epinephrine administration	25 (7.1)	10 (6.2)	15 (8.0)	.66
IVF use, no. (%)	57 (16.3)	26 (16.0)	31 (16.5)	.99
Preexisting eczema, no. (%)	161 (46.0)	67 (41.4)	94 (50.0)	.13
Prior allergy visit, no. (%)	99 (28.3)	24 (14.8)	75 (39.9)	<.001
Food triggers, no. (%)				.17
Peanut	47 (13.4)	17 (10.5)	30 (16.0)	.18
Milk	33 (9.4)	29 (17.9)	4 (2.1)	<.001
Eggs	27 (7.7)	16 (9.9)	11 (5.9)	.23
Tree nut and seeds	22 (6.3)	6 (3.7)	16 (8.5)	.10
Seafood	5 (1.4)	1 (0.6)	4 (2.1)	.46
Meat	2 (0.6)	2 (1.2)	0 (0.0)	.41
Fruit and vegetables	1 (0.3)	0 (0.0)	1 (0.5)	.99
Anaphylaxis (FIA), no. (%)	182 (52.0)	64 (39.5)	118 (62.8)	<.001
Refractory anaphylactic reactions, no. (%)	88 (25.1)	45 (27.8)	43 (22.9)	.35

*IVF, In vitro* fertilization.

∗ Defined as documented on the medication list 1 year before ER encounter.

**Fig 2:**
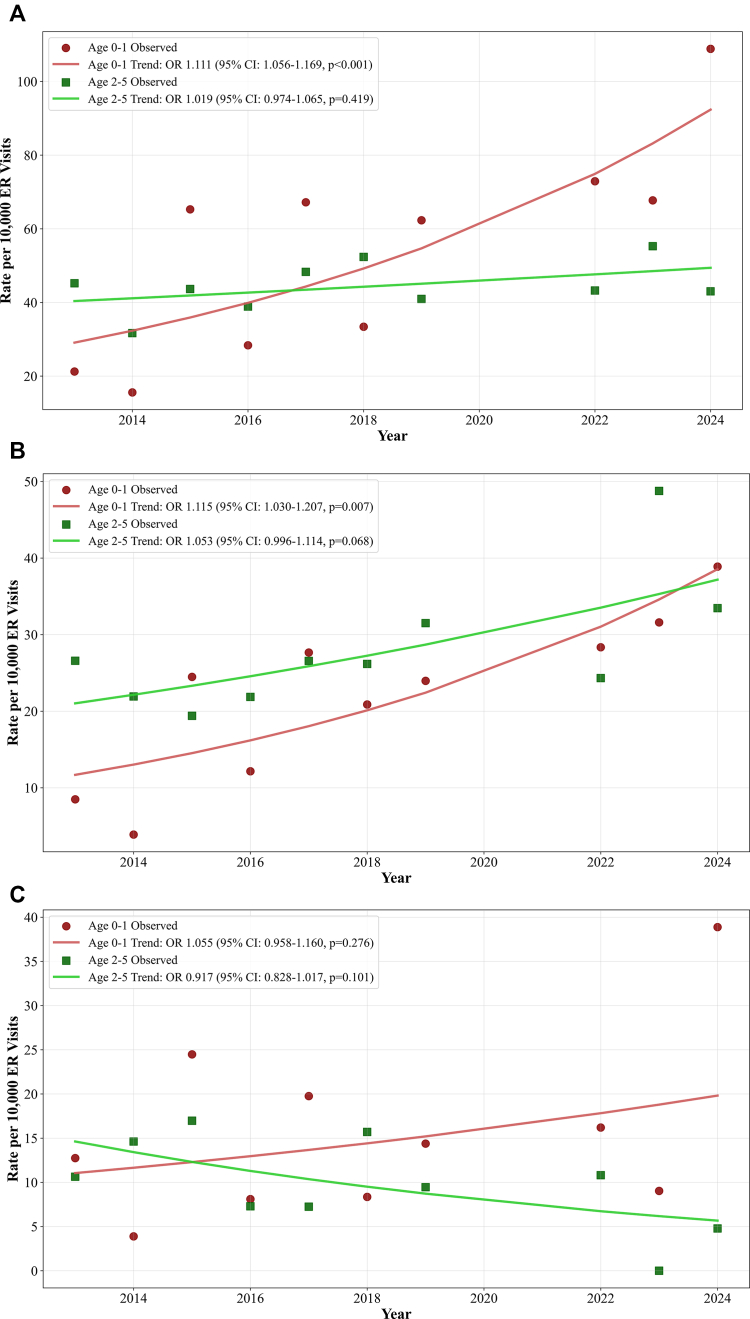
Trends in food-induced ER visits per 10,000 encounters among children aged 0 to 5 years from 2013-2024 with the coronavirus disease 2019 (COVID-19) pandemic years (2020-2021) excluded. **A,** Food-induced reactions. **B-C,** Food-induced anaphylaxis (**B**) and refractory anaphylactic reactions (**C**). Rates are stratified by age group (*red indicates age 0-1 year; green indicates age 2-5 years*). Solid lines represent logistic regression trends derived by using marginalization standardization. Corresponding average odds ratios per year with 95% CIs and *P* values are shown in the figure legend boxes.

Annual FIR-related visit rates increased significantly over time among infants aged 0 to 1 year (OR = 1.111 per year [95% CI = 1.056-1.169] [*P* < .001]), rising from 21.2 to 108.9 per 10,000 ER visits in the same age group (ie, a 414% increase over the course of the study period). In contrast, FIR rates remained stable in children aged 2 to 5 years (OR = 1.019 per year [95% CI = 0.974-1.065] [*P* = .419]) during the same time interval (Fig 2, *A*). FIA rates in infants (aged 0-1 years) rose from 8.5 to 38.9 per 10,000 (a 358% increase [OR = 1.115 per year (95% CI = 1.030-1.207)] [*P* = .007]), whereas rates in children aged 2 to 5 years trended upward but did not reach statistical significance (16.4-20.7 per 10,000 [OR = 1.053 per year, 95% CI = 0.996-1.114] [*P* = .068]) (Fig 2, *B*). Refractory anaphylactic reaction rates did not change significantly in either infants (OR = 1.055 per year [95% CI = 0.958-1.160] [*P* = .276]) or children aged 2 to 5 years (OR = 0.917 per year [95% CI = 0.828-1.017] [*P* = .101]) (Fig 2, *C*).

**Fig 3:**
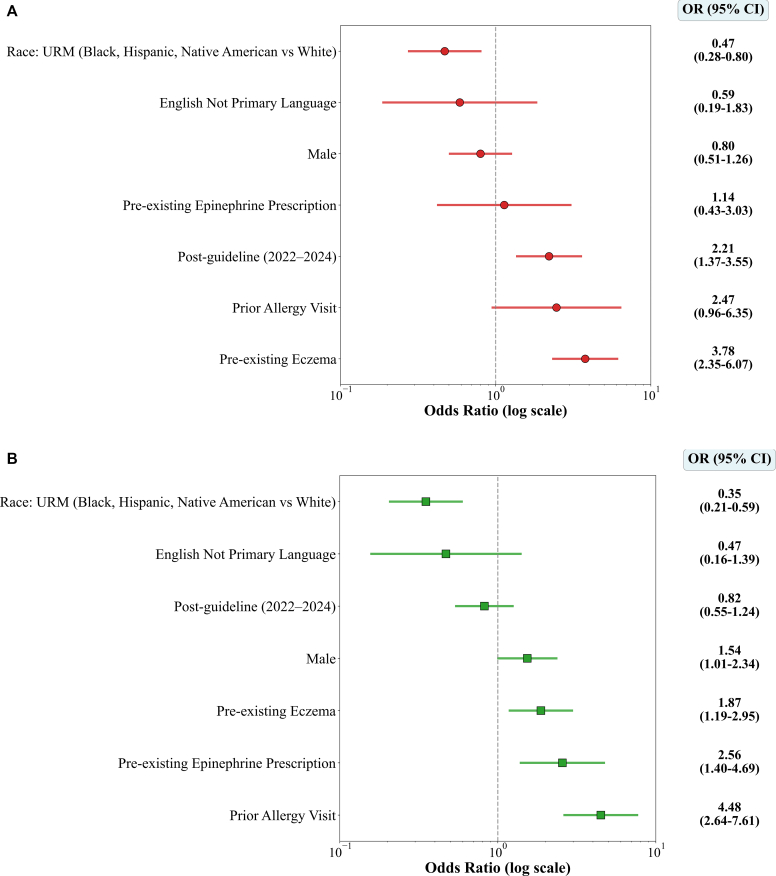
Predictors of FIRs by age group: age 0 to 1 years (**A**) and age 2 to 5 years (**B**). Odds ratios and 95% CIs are displayed on a log scale. Infants showed increased odds of reactions during the postguideline period (2022-2024), whereas children aged 2 to 5 years did not. Eczema and prior allergy visits were significant predictors in both groups; URM status was associated with lower odds.

To delineate changes across interval time periods, we next performed adjusted logistic regression comparing FIRs between the preguideline and postguideline periods. The models were adjusted for clinical and demographic covariates presumed to affect FIRs, including preexisting eczema, sex, race/underrepresented minority (URM) status, prior allergy visits, and prior epinephrine prescription. There were 96 FIR encounters in 91 patients during the preguideline period (2013-2016) and 89 FIR encounters in 88 patients during the postguideline period (2022-2024). Among infants, FIRs occurred significantly more during the postguideline period than during the preguideline period (OR = 2.21 [95% CI = 1.40-3.48]), suggesting an age-specific shift that presumably reflects the national guideline-based changes in infant food allergen introduction (Fig 3, *A*). No significant difference in association was observed in the 2- to 5-year age group between these 2 time intervals (OR = 0.82 [95% CI = 0.55-1.23]) (Fig 3, *B*), although this analysis should be interpreted with caution given the small sample size (N = 88).

Additional predictors of FIRs in infants included preexisting eczema (OR = 3.78 [95% CI = 2.35-6.07]) with lower odds in URM groups (OR = 0.47 [95% CI = 0.28-0.80]) (Fig 3, *A*). In the 2-year to 5-year age group, FIR was associated with male sex (OR = 1.54 [95% CI = 1.01-2.34]), eczema (OR = 1.87 [CI = 1.19-2.95]), prior allergy visits (OR = 4.48 [95% CI = 2.64-7.61]), and preexisting epinephrine prescriptions (OR = 2.56 [95% CI = 1.40-4.69]) and lower odds among URM groups (OR = 0.35 [95% CI = 0.21-0.59]) (Fig 3, *B*).

In summary, between 2013 and 2024, the number of ER encounters for FIRs and FIA rose significantly in infants (aged 0-1 years) but not in children aged 2 to 5 years. Adjusted logistic regression showed that infants had more than twice the odds of presenting with a FIR in the postguideline period 2022-2024 than in 2013-2016. Trends in FIA rates among children aged 2 to 5 years showed a nonsignificant increase. Given the limited sample size, larger or ongoing observational studies will be needed to determine whether this represents a true signal. Reassuringly, refractory anaphylactic reaction rates have seemingly remained stable in both age groups. Our results are consistent with those of Chow et al, who showed that Australian infant ER presentations of food allergy increased after the 2016 guideline update, without a similar rise in older children.^11^^,^^12^

We presume that these findings reflect new national recommendations of early allergen introduction in infants. Although this recommendation unquestionably decreases the risk of developing food allergies later in life, it is important to recognize that a proportion of these children will have already been sensitized and clinically reactive at the time of the first feed. Our observation that infant ER visits for a FIR increased during the postguideline implementation period highlights the need to identify those at high risk before the initiation of food allergen introduction. Among these are infants with other comorbidities such as severe eczema and/or gastrointestinal complaints.^8^ This population may require closer observation and targeted anticipatory guidance, including detailed advice on the recognition, assessment, and management of food-related reactions.^13^

Despite rising FIR and FIA rates, rates of refractory anaphylactic reactions have remained stable in infants and have decreased slightly in children aged 2 to 5 years (Fig 2, *C*). This also was consistent with findings from Australia, which showed declining epinephrine use and hospital admissions in infants following guideline implementation.^11^ The rise in FIR and FIA encounters may reflect an increase in mild reactions during infancy, with caregiver uncertainty or limited confidence in managing reactions at home contributing to more frequent emergency visits for self-limited symptoms.^13^ Increased exposure risk from early allergen introduction, along with disparities in caregiver education and access to nonurgent allergy care, may further contribute to this pattern.^14^ Improved caregiver education and overall awareness may help reduce unnecessary ER use for many reactions.^4^^,^^6^

A key limitation of this study is the assumption of widespread adoption of early food introduction guidelines. In a 2021 US survey, only 17% of caregivers reported introducing peanut before age 7 months and 59% by age 12 months.^15^ In contrast, Australian introduction rose from 28% to 89% following updated guidance in 2016.^16^ To mitigate this variability, we focused on a postguideline period (2022-2024) after the last recommendation change in 2021. Although adoption may be higher in academic or urban settings such as ours, actual uptake in this population remains unmeasured. Additionally, time block stratification reduced effective sample sizes in the comparisons between guideline periods; nevertheless, the postguideline effect remained significant, suggesting robust age-specific effects. As adoption improves over time, more studies will be needed to determine not only the number of infants having reactions but also the number of those who successfully tolerate early allergen introduction. Studies that capture infants exposed to early introduction via population-based surveys and ongoing surveillance of reaction rates in older children are warranted.

This study is also limited by the use of a de-identified administrative database, which lacks clinical notes and contains discrete electronic health record data only. Although we excluded drug- and venom-related codes through manually reviewed diagnoses, anaphylaxis codes remain broad and may capture nonfood triggers. We are reassured that most anaphylaxis in this age group is food related.^17^^,^^18^ We were also unable to distinguish IgE- from non–IgE-mediated reactions owing to limited coding granularity.^19^ Given this constraint, we chose to examine all food-induced reactions rather than restrict our analysis to anaphylaxis alone.

In summary, this is the first exploratory US study to evaluate food allergy–related ER trends following changes in the national guidelines regarding early allergen introduction. We observed increased infant ER visits for FIR and FIA, reflecting earlier food allergen introduction. As guideline adoption expands, anticipatory guidance is necessary to prepare families and caretakers in the event of food reactions. Future studies should evaluate adoption and parental attitudes of early allergen introduction, as well as whether guidance for high-risk infants reduces ER utilization.

For additional details on methods, see the Supplementary Methods in the Online Repository at www.jaci-global.org.

## Disclosure statement

Supported by the National Institutes of Health/National Center for Advancing Translational Science and the University of California Los Angeles Clinical and Translational Science Institute (grant UL1TR001881).

Disclosure of potential conflict of interest. The authors declare that they have no relevant conflicts of interest.
