# Supplementary material for: Rising trends in infant ER encounters for food-induced allergic reactions in the era of early allergenic food introduction
Source: J Allergy Clin Immunol Glob. 2025 Dec 22;5(2):100637. doi: 10.1016/j.jacig.2025.100637 (PMC12809485; doi:10.1016/j.jacig.2025.100637)
Supplement: Supplementary Material [file mmc1.docx]

**Online Repository**

**Methods**

Case and Clinical Ascertainment

We queried the UCLA de-identified data repository for all emergency department (ED) encounters among patients aged 0–5 years from January 1, 2013, through December 31, 2024 (Figure 1). Food-induced reactions (FIR) were identified using ICD codes for food allergy (T78.1*, Z91.01*, V15.01–V15.05) and food-induced anaphylaxis (FIA) (T78.0*, T78.2*, 995.6, 995.0), based on encounter diagnoses, admission fields, or billing claims. Encounters with drug- or venom-related codes (T63*, Z91.03*, T80.5*, T88.6*, 989.5, V15.06–V15.09, 995.2) were excluded. Because anaphylaxis at this age group is primarily food-induced, we assumed that general anaphylaxis code was likely due to food triggers.(18, 19) Additionally, diagnosis text was manually reviewed to exclude clearly non-food-related visits.

Encounters were stratified by age into two groups: aged infants (0–1 years) and those aged 2–5 years. FIA was defined by either anaphylaxis-related diagnosis codes or epinephrine administration within the ED. Refractory anaphylaxis reactions were defined as requiring multiple epinephrine doses or resulting in hospital admission. Specific food triggers (e.g., peanut, milk, egg) were identified using food-specific ICD codes.

Demographics were recorded at the time of the ED visit. Eczema was identified using ICD codes (L20*, 691*, L21*, L30*) from the index encounter, prior visits, or the problem list. Pre-existing epinephrine prescriptions were defined by the presence of an active injectable epinephrine order within one year before the ED visit. Prior allergy evaluations were identified by any outpatient encounter with an allergy/immunology provider.

All data were obtained from the UCLA Discovery Data Repository, a de-identified limited dataset without direct patient identifiers; thus, this study was exempt from institutional review board (IRB) review.

Annualized Trend Analysis

Yearly rates of FIR, FIA, and severe reactions were calculated per 10,000 ED visits. For each year, the number of qualifying events was divided by the total number of ED visits within each age group (0–1 and 2–5 years). Annual trends were evaluated using logistic regression. Odds ratios (OR) per year with 95% confidence intervals (CI) and p-values were reported. Predicted rates were estimated using marginal standardization for visualization. Due to substantial shifts in ED utilization during the COVID-19 pandemic, data from 2020 and 2021 were excluded from trend analyses.

Multivariable Regression

To assess the impact of national food allergy prevention guidelines, we performed logistic regression stratified by age group (0–1 and 2–5 years). The primary exposure was encounter year group, comparing the post-guideline period (2022–2024) to a pre-guideline reference period (2013–2016). These timeframes were selected to account for delayed uptake following major U.S. guideline publications. Models were adjusted for a priori selected clinical variables (eczema, prior allergy evaluation, and pre-existing epinephrine prescription) and demographic factors, including sex and underrepresented minority (URM) status, defined as Black, Hispanic/Latino, or Native American. Standard errors were clustered by patients to account for potential correlation from patients with multiple encounters.

Analyses were performed in Python 3.9.13 using the following packages: pandas, NumPy, statsmodels, scipy, and matplotlib. Statistical significance was determined using a two-sided alpha level of 0.05.
